# Hepatitis C among Egyptian Patients Referred for Bone Marrow Examination: Seroprevalence and Analysis of Hematological Findings

**DOI:** 10.1155/2014/549716

**Published:** 2014-04-10

**Authors:** Somaia Mohammed Mousa

**Affiliations:** Clinical Pathology Department, Kasr Al-Ainy School of Medicine, Cairo University, P.O. Box 99, Manial El-Roda, Cairo 11553, Egypt

## Abstract

Hepatitis C is a significant public health problem in Egypt where the highest prevalence (14.7%) of hepatitis C virus (HCV) exists. HCV prevalence is even higher among clinical populations and groups at risk of exposure to infection. Chronic HCV infection is associated with several hematological complications that may necessitate bone marrow (BM) examination. The aim of this study is to estimate HCV prevalence among patients referred for BM examination and to explore hematological and BM findings among HCV positive patients. One hundred adult patients referred for BM examination were included in the study and screened for HCV antibodies. Patients' clinical, hematological, and BM findings were recorded. The seroprevalence of HCV among patients referred for BM examination was 42%. The most common indication for BM examination among HCV positive patients was peripheral cytopenias (88.1%). The most common cytopenia detected was thrombocytopenia (85.7%). The most common diagnosis among HCV positive patients was hypersplenism (52.4%) followed by B-lymphoproliferative disorders (19%) and then immune thrombocytopenic purpura (11.9%). In conclusion, HCV prevalence among patients referred for BM examination is higher than that estimated in the general population. Patients with unexplained peripheral cytopenias should be tested for HCV.

## 1. Introduction

Hepatitis C virus (HCV) infection is a major health problem in Egypt. Egypt has the highest HCV prevalence worldwide [1]. The estimated prevalence of HCV in Egypt is 14.7% among general population in the year 2008 [2]. HCV prevalence is even higher among hospitalized patients and special clinical populations [3]. Most HCV-infected patients have no hepatic symptoms, and extrahepatic syndromes may be the reason for which they seek medical advice [4]. Chronic HCV infection is associated with several benign as well as malignant hematological complications. HCV is a lymphotropic virus [5] that could be associated with lymphoproliferative disorders including B-cell non-Hodgkin's lymphoma (NHL) and monoclonal gammopathies [6]. Peripheral cytopenias are common hematological derangements that are associated with HCV [7]. They occur because of peripheral sequestration in the spleen (hypersplenism), peripheral destruction by immune mechanism, or antiviral therapy [8]. Patients with HCV infection may be referred for bone marrow (BM) examination due to any of these complications. The aim of this work is to study the prevalence of HCV infection among Egyptian patients referred for BM examination and to explore hematological and BM findings among HCV positive patients.

## 2. Patients and Methods

The study included 100 patients referred to the Hematology Unit, Clinical Pathology Department, Kasr Al-Ainy School of Medicine, Cairo University. Patients were referred from different hospital departments for BM examination due to various indications. Non-Egyptians and patients below the age of 14 years were excluded from the study. The first 100 patients who met the study criteria were consecutively included in the study. Patients were subjected to history taking and clinical examination. The presence of organomegaly was determined by abdominal ultrasound. Complete blood count and fresh blood smear examination were done. BM was aspirated from the posterior iliac crest under local anesthesia with standard aseptic conditions. BM aspirate (BMA) smears were stained with Leishman stain for morphological examination. Patients were screened for HCV antibodies by enzyme linked immunosorbent assay. According to our institutional guidelines, cytopenias were defined as hemoglobin concentration less than 13 g/dL in males and 12 g/dL in females [9], total leukocyte count less than 4 × 10^3^/*μ*L, and platelet count less than 150 × 10^3^/*μ*L [10].

The patients' clinical and haematological parameters were recorded and the data was tabulated. Numerical data were expressed as mean ± standard deviation (SD) and compared by Student's *t*-test. Qualitative data were expressed as frequency and percentage and compared by Chi-square test. *P* < 0.05 was considered significant.

## 3. Results

One hundred patients referred to Hematology Unit, Clinical Pathology Department, for BM examination are included in the study and screened for HCV antibodies. The age of the patients ranged from 17 to 73 years with a mean of 42.92 ± 14.47 years and fifty patients were males (50%). Screening of patients for HCV antibodies revealed that 42 patients (42%) were HCV positive.

Clinical and hematological criteria of HCV positive and HCV negative patients are compared in Table 1.

Splenomegaly was significantly more frequent among HCV positive patients.

HCV positive patients had a significantly lower platelet count compared to HCV negative patients.

Among HCV positive patients, only one thalassemic patient and one patient with chronic renal failure on hemodialysis were included; they were referred for bone marrow examination due to hematological derangements. Other patients (95%) did not belong to any special clinical population groups that are known to have high HCV prevalence like hemophilia, thalassemia, and hemodialysis.

The most common indication for BMA among HCV positive patients was peripheral cytopenias that were seen in 37 patients (88.1%). Sixteen patients (38.1%) suffered from pancytopenia, 11 patients (26.2%) had bicytopenia in the form of anemia and thrombocytopenia, 3 patients (7.1%) had combined leucopenia and thrombocytopenia, 3 patients (7.1%) had isolated anemia, 3 patients (7.1%) had isolated thrombocytopenia, and 1 patient (2.4%) had isolated leucopenia. Four patients (9.5%) were diagnosed as B-NHL and had combined BMA/biopsy examination for staging and 1 patient (2.4%) performed BM aspiration as a part of acute leukemia work-up.

NHL patients suffered from hematological derangements in the form of pancytopenia (2 patients), combined anemia and leucopenia (1 patient), and isolated anemia (1 patient). The acute leukemia patient presented with pancytopenia. So, the most common cytopenia detected among HCV positive patients was thrombocytopenia (85.7%), followed by anemia (83.3%) and then leucopenia (57%).

Table 2 shows the diagnoses of HCV positive patients based on BM examination. Out of the four patients with reactive marrow hyperplasia, two patients were previously diagnosed as NHL with lymph node biopsy but had no BM infiltration. The most common diagnosis among HCV positive patients was hypersplenism (52.4%), followed by B-lymphoproliferative disorders (19%), and then immune thrombocytopenic purpura (ITP) (11.9%). Hypersplenism was presented as pancytopenia in 12 patients (54.5%), combined anemia and thrombocytopenia in 4 patients (18.2%), combined leucopenia and thrombocytopenia in 3 patients (13.6%), isolated thrombocytopenia in 2 patients (9.1%), and isolated anemia in 1 patient (4.5%). Lymphoproliferative disorders cases were 4 patients (9.5%) with B-NHL, 3 patients (7.1%) with Waldenstrom macroglobulinemia, and 1 (2.4%) multiple myeloma patient.

## 4. Discussion

To our knowledge, our study offers the first report on seroprevalence of HCV among patients referred for BM examination. We detected HCV antibodies in 42% of this special group of patients, which is much higher than that reported in the general population in Egypt (14.7%) [2]. Similarly, many Egyptian studies found that HCV prevalence was high across all special clinical population groups like hemodialysis patients (35%) [11], hemophilic children (40%) [12], multitransfused thalassemic patients (40.5%) [13], and NHL patients (43%) [14]. These groups of patients have an increased risk of exposure to HCV infection because of hospitalization, repeated blood transfusions, invasive procedures, injections, or shared dialysis machines. In the current study, most HCV positive patients (95%) did not belong to any of these groups. Higher HCV prevalence among our patients may be related to HCV complications that necessitate BM examination rather than higher risk of patients' exposure to HCV. High prevalence of HCV among patients referred for BM examination in our setting raises concerns about precautions that should be taken for protection of health care professionals and for prevention of transmission of infection. It is recommended for healthcare workers to consider each blood or body fluid sample as potentially infectious [15] and to practice standard infection control precautions and basic hand hygiene (including double gloving) while performing exposure prone interventions for patients [16].

In the current study, the commonest indication for BMA in HCV positive patients was peripheral cytopenias (88.1%). Pancytopenia was seen in 38.1% of patients and bicytopenia in 33.3% of cases. In this context, we could recommend screening of patients suffering from peripheral cytopenias for HCV prior to BM examination as their hematological derangements could be explained by HCV. Only two previous studies have investigated BM findings among HCV positive patients. In the study performed by Klco and colleagues, peripheral cytopenias were the commonest indication (83%) for BM examination in HCV positive patients, with pancytopenia seen in 12.8% of cases and bicytopenia in 44.7% [17]. Anwar et al. studied BM findings among exclusively HCV positive patients with hematological derangements and found pancytopenia in 53.3% of cases and bicytopenia in 18.6% of patients [18].

In this study, the most common cytopenia detected was thrombocytopenia (85.7%) and platelet count was significantly lower among HCV positive compared to HCV negative patients. Several studies reveled that among the haematological derangements in chronic HCV infection, the decrease of platelet number seems to be the most common [19–21].

The pathogenesis of thrombocytopenia among chronic HCV-infected patients is multifactorial. Possible causes include sequestration because of hypersplenism secondary to portal hypertension and splenomegaly, bone marrow suppression either by HCV directly or by antiviral treatment, immune-mediated platelet destruction, and impaired thrombopoietin production resulting from hepatocellular damage [8]. A new thrombopoietin receptor agonist (eltrombopag) is now approved in the United States for treatment of thrombocytopenia in patients with chronic HCV in order to increase the platelet count to a level that allows the initiation and maintenance of antiviral therapy [22]. The exact cause of peripheral cytopenias is not properly investigated in this study; however, 76% of patients had splenomegaly and more than half of patients were diagnosed as hypersplenism. In concordance with our results, 77% of HCV positive patients referred for BM examination in the two previously mentioned studies had splenomegaly [17, 18] with hypersplenism being the commonest diagnosis [18].

The second common diagnosis among our group of HCV positive patients was B-lymphoproliferative disorders (19%) that were also detected in 10.7% of HCV positive patients investigated by Klco et al. [17]. The association between HCV and B-lymphoproliferative disorders is well known and attributed to a complex, multistep, multifactorial process, probably based on sustained B-lymphocyte activation and the inhibition of B-lymphocyte apoptosis within a background of predisposing genetic factors [6].

In the current study, the third common diagnosis was ITP (11.9%). ITP was detected in 22.7% of HCV positive patients investigated by Anwar et al. [18]. Secondary ITP can occur in association with HCV infection and the recent American Society of Hematology practice guideline for ITP recommends HCV testing for all patients with acute ITP, because treatment of HCV may alter the course of secondary ITP [23].

## 5. Conclusion

HCV prevalence among patients referred for BM examination in our setting is high. Health care workers should take adequate infection control precautions while performing any patients' intervention like BM aspirate/biopsy. Any patient with unexplained cytopenias or recently diagnosed as ITP should be tested for HCV. This study is limited by lack of data regarding duration of HCV infection, level of HCV viremia, antiviral therapy received, and coinfection with other viruses.

## Figures and Tables

**Table 1:** Clinical and hematological parameters of HCV positive (*n* = 42) and HCV negative patients (*n* = 58).

Parameter	HCV positive patients	HCV negative patients	*P*
Age (mean ± SD years)	47.38 ± 11.64	39.91 ± 15.47	0.01*
Males *n* (%)	21 (50)	29 (50)	1.00
Splenomegaly *n* (%)	32 (76)	30 (52)	0.02*
Hepatomegaly *n* (%)	15 (36)	16 (28)	0.52
Lymphadenopathy *n* (%)	4 (10)	12 (21)	0.22
Hb% (mean ± SD g/dL)	9.73 ± 2.50	8.91 ± 3.00	0.15
RBCs (mean ± SD ×10^6^/*μ*L)	3.45 ± 0.99	3.17 ± 1.17	0.21
MCV (mean ± SD fL)	85.61 ± 10.89	85.16 ± 9.45	0.83
MCH (mean ± SD pg)	28.47 ± 4.69	28.65 ± 3.59	0.83
TLC (mean ± SD ×10^3^/*μ*L)	5.02 ± 3.64	8.92 ± 19.37	0.20
Platelets (mean ± SD ×10^3^/*μ*L)	72.71 ± 63.41	138.48 ± 187.60	0.03*

HCV: hepatitis C virus, Hb: hemoglobin, RBCs: red blood cells, MCV: mean corpuscular volume, fL: femtoliter, MCH: mean corpuscular hemoglobin, pg: picogram, TLC: total leukocyte count, *a statistically significant value.

**Table 2:** Diagnoses of HCV positive patients based on BM examination (*n* = 42).

Diagnosis	*n* (%)
Hypersplenism	22 (52.4%)
ITP	5 (11.9%)
Reactive hyperplasia	4 (9.5%)
Waldenstrom macroglobulinemia	3 (7.1%)
Aplastic anemia	3 (7.1%)
NHL (stage IV)	2 (4.8%)
Multiple myeloma	1 (2.4%)
Megaloblastic anemia	1 (2.4%)
Acute leukemia	1 (2.4%)

HCV: hepatitis C virus, ITP: immune thrombocytopenic purpura, and NHL: non-Hodgkin's lymphoma.
